# In situ HER2 RNA expression as a predictor of pathologic complete response of HER2-positive breast cancer patients receiving neoadjuvant chemotherapy and anti-HER2 targeted treatment

**DOI:** 10.1186/s13058-024-01852-3

**Published:** 2024-06-12

**Authors:** Huang-Chun Lien, Chiao Lo, Yi-Hsuang Lee, Po-Hang Lin, Ming-Yang Wang, Wen-Hung Kuo, Li-Wei Tsai, Yen-Shen Lu, Hsiang-Wei Hu, Yu-Chia Li, Chiun-Sheng Huang

**Affiliations:** 1 Department of Pathology, National Taiwan University Hospital, Taipei, Taiwan; 2 Graduate Institute of Pathology, National Taiwan University, Taipei, Taiwan; 3 Department of Surgery, National Taiwan University Hospital, Cancer Center Branch. No.57, Ln. 155, Sec. 3, Keelung Rd., Da’an Dist., Taipei City, 106 Taiwan; 4 Department of Medical Genetics, National Taiwan University Hospital, Taipei, Taiwan; 5 Department of Oncology, National Taiwan University Hospital, Taipei, Taiwan

**Keywords:** Breast cancer, In situ HER2 RNA expression, RNAscope, Immunohistochemistry, Fluorescence in situ hybridization, Neoadjuvant chemotherapy and anti-HER2 target therapy, Pathological complete response

## Abstract

**Background:**

Immunohistochemistry (IHC) and in situ hybridization (ISH) remain standard biomarkers for therapeutic decisions in human epidermal growth factor 2 (HER2)-positive breast cancers (BCs); however, they are insufficient to explain the heterogeneous anti-HER2 response.

**Methods:**

We aimed to investigate the correlation of in situ HER2 RNA expression (isHRE), using RNAscope, with HER2 biomarkers and the impact of isHRE on the pathological complete response (pCR) rates of 278 patients with HER2 IHC/fluorescence ISH (FISH)-positive BC receiving neoadjuvant chemotherapy and anti-HER2 targeted treatment (NCTT).

**Results:**

We validated HER2 RNAscope scoring as a semiquantitative method to determine isHRE and showed a positive correlation between RNAscope scores and pCR rates, with particularly different rates between patients with a score of 5 versus 1–4 BCs (66.7% vs. 15.9%, *p* < 0.0001). There were higher RNAscope scores and pCR rates in patients with HER2 IHC 3 + versus IHC 2+/FISH + BCs and HER2 RNAscope scores and pCR rates showed similar non-linear positive correlations with HER2 copy numbers and HER2/centromere 17 ratios. Moreover, in each HER2-positive IHC/FISH category, higher pCR rates were observed in patients with RNAscope scores of 5 versus 1–4 BC. Patients achieving pCR had BCs with notably higher HER2 RNAscope scores. Multivariate analysis identified HER2 RNAscope 5 as a strong pCR predictor [odds ratio = 10.865, *p* < 0.001]. The combined impact of multivariate analysis-defined pCR predictors demonstrated that a higher pCR rate was observed in patients with a score of 5 versus a score of 1–4 BCs regardless of the status of hormone receptor and mono-or dual anti-HER2 blockade.

**Concusions:**

Our results demonstrated that high isHRE (RNAscope score 5) is a strong pCR predictor in patients with HER2-positive BCs receiving NCTT, highlighting the complementary role of isHRE in stratifying HER2 status in tissue. Such stratification is relevant to anti-HER2 therapeutic efficacy, particularly using the cutoff of score 1–4 versus 5.

**Supplementary Information:**

The online version contains supplementary material available at 10.1186/s13058-024-01852-3.

## Introduction

Approximately 15% of invasive breast cancers (BCs) are human epidermal growth factor receptor 2 (HER2)-positive, defined by HER2 gene amplification or protein overexpression [1]. Such tumors are sensitive to anti-HER2 targeted therapy [2, 3] and, currently, a combination of chemotherapy and anti-HER2 targeted therapy is considered the standard treatment for HER2-positive BCs, both in adjuvant and neoadjuvant setting [4]. The HER2 expression status is critical for selecting patients with BC eligible for HER2-targeted therapies. Currently, the determination of HER2 gene status is based on immunohistochemistry (IHC) and in situ hybridization (ISH), and HER2-positive BC is defined as either IHC 3 + or IHC 2+/ISH+.

The response of patients with HER2-positive BC to HER2-targeted therapy is significant; however, the response is not universal, and a proportion of patients with HER2-positive BC do not respond [5–7]. In addition, conflicting data exist in the literature regarding the relationship between anti-HER2 therapeutic response and HER2 protein expression or HER2 ISH parameters, including HER2 and centromeric 17 (CEP17) copy numbers and HER2 /CEP17 copy number ratios, in both adjuvant and neoadjuvant settings [7–16]. Neoadjuvant HER2-directed therapy provides an opportunity to determine the in vivo response to therapy, as patients who achieve a pathological complete response (pCR) can anticipate a higher probability of avoiding both disease recurrence and death from BC [17–19]. However, pCR rates range from approximately 20–60% in patients with HER2-positive BCs [7, 20]. These findings suggest that although amplification and/or overexpression of HER2 remains the only biomarker for therapeutic decisions, it is insufficient to explain the heterogeneous response to anti-HER2 therapy [5]. This underscores the need for novel HER2 biomarkers, in addition to the current standards of IHC and ISH, to better predict the efficacy of anti-HER2 therapy.

RNAscope, an ISH-based analysis of RNA has been used to analyze RNA in single cells with single-molecule sensitivity [21]. It has been shown to be a valid alternative for determining HER2 status in tissues in an automated setting and has been demonstrated to further stratify HER2 levels in HER2-equivocal tumor cases [21, 22]. RNA sequencing (RNA-seq) in a secondary analysis of the NeoALTTO randomized clinical trial showed that HER2 expression was the most significant predictor of pCR [5]. However, the correlation between in situ HER2 RNA expression (isHRE), which evaluates HER2 RNA expression in tissue sections, and the status of HER2 IHC and ISH in HER2-positive BCs and their impact on anti-HER2 therapeutic efficacy remains unexplored. In this study, we analyzed isHREs using RNAscope in 278 patients with HER2-positive BC receiving neoadjuvant chemotherapy and anti-HER2 targeted therapy (NCTT). Our goal was to investigate the role of isHRE as a HER2 biomarker and its potential impact on anti-HER2 therapeutic efficacy in a neoadjuvant setting.

## Materials and methods

### Patients and tumor samples

We retrieved formalin-fixed, paraffin-embedded (FFPE) specimens from the Department of Pathology of National Taiwan University Hospital (Taipei, Taiwan), collected between 2011 and 2019, from 278 patients that had HER2-positive BCs and received NCTT. These HER2-positive BCs were either HER2 IHC 3 + or IHC 2+/FISH + based on the version of American Society of Clinical Oncology (ASCO)/ College of American Pathologists (CAP) HER2 guideline upon diagnosis [23–25]. All the 7 HER2 IHC2+/FISH group 2 cases were diagnosed in 2014–2016, and were considered as HER2-positive BCs based on the 2013 ASCO/CAP criteria. An additional 35 BC specimen, including 14 HER2-negative cases (IHC 2+/FISH-, IHC 1 + and 0) and 21 IHC 3 + cases, were included in the validation for HER2 RNAscope. Clinicopathological information were obtained from the medical charts. Estrogen receptor (ER) or progesterone receptor (PR) positivity was defined using a 1% cutoff. Hormone receptor (HR) positivity and negativity was defined as positivity for either ER or PR or negativity for both ER and PR, respectively. The NCTT treatment subgroups included epirubicin (E) and cyclophosphamide (C) ± fluorouracil (F), followed by taxotere or taxol (T) and herceptin (H) ± pertuxumab (P) [EC(F) to TH(P)]; T and H ± P followed by E and C ± F [TH(P) to EC(F)]; T and H ± P [TH(P)]; and T, carboplatin (Carbo), and H ± P [TCarboH(P)]. All the 278 patients received post-NCTT mastectomy. Thirty-nine patients did not receive post-NCTT lymph node sampling: 34 patients had no axillary lymph node metastasis on pre-NCTT lymph node sampling; three and one patients had micrometastasis and macrometastasis on only one of the pre-NCTT sampled lymph nodes, respectively. All the 38 patients had no clinical evidence of post-NCTT axillary lymph node metastasis. The remaining one patient had pre-NCTT lymph node metastasis and post-NCTT ypT2, but refused post-NCTT lymph node sampling. pCR was defined as no residual invasive carcinoma in the breast and axillary lymph nodes (ypT0/is, ypN0) upon surgical resection. The level of tumor infiltrating lymphocytes (TIL) were evaluated on available slides, based on the published recommendation [26].

### HER2 FISH and HER2 RNA in situ hybridization

For HER2 IHC 2 + BCs, the results of reflex HER2 FISH were collected from the pathology reports. For all HER2 IHC 3 + cases, additional FISH tests were performed using the PathVysion HER2 DNA Probe Kit (Abbott, Abbott Park, IL, USA). The results were recorded according to the 2018 ASCO/CAP guideline [25]. HER2 ISH was performed using an RNAscope FFPE 2.5 kit (Advanced Cell Diagnostics, Inc., Hayward, CA, USA). Briefly, FFPE section slides were treated with citrate buffer and protease before sequential hybridization with HER2 probes (RNAscope Probe-Hs-ERBB2), preamplifiers, amplifiers, and label probes. Hybridization signals were detected by 3,3’-diaminobenzidine (DAB) staining. The slides were visualized by microscopy (400× magnification) and at least 40 tumor cells were evaluated for each case. isHRE was semiquantitatively categorized into six RNAscope scores based on a modification of previously described criteria: [21, 22] score 0 (< 1 individual dots/tumor cells), score 1 (1–3 individual dots/tumor cells), score 2 (4–9 individual dots/tumor cells), score 3 (10–15 individual dots/tumor cells), score 4 (> 15 individual dots/tumor cells and ≤ 50% clustered dots), and score 5 (> 15 individual dots/tumor cells and > 50% clustered dots or diffuse dense cytoplasmic signal). For all cases with score ≤ 2, RNAscope with a probe for the housekeeping gene peptidylprolyl isomerase B (PPIB) (Advanced Cell Diagnostics, Inc.) were performed to evaluate RNA integrity. The PPIB score was the same as that for HER2. Cases with PPIB score ≤ 1 were considered to have degraded RNA and were excluded for subsequent analysis. All slides were independently scored by two breast pathologists (HCL and YHL) who had no knowledge of the clinicopathological features or treatment results at the time of scoring. An additional pathologist (SWH) reviewed cases with discrepant scores and a final consensus was reached and recorded.

### Quantitative reverse transcriptase polymerase chain reaction and RNA-seq

For each case, tumor sections from six slides (10 μm) were macrodissected and guided by the corresponding hematoxylin and eosin staining. RNA was extracted using the RNeasy FFPE KIT (QIAGEN, Hilden, Germany). RNA was reverse-transcribed into cDNA using the MMLV Reverse Transcriptase Kit (Protech, Taipei, Taiwan). Quantitative reverse transcriptase polymerase chain reaction (qRT-PCR) was performed in triplicate on a QuanStudio 7 Flex Real-time PCR using the Power SYBR Green PCR Master Mix (Applied Biosystems, Foster City, CA, USA). The quantitative values were calculated using the delta-delta cycle threshold (Ct) method. HER2 expression in all samples was normalized to that of glyceraldehyde-3-phosphate dehydrogenase (GAPDH). The primers used in this study are listed (Supplementary Table 1). A total of 150 cases enrolled in a study (unpublished) involving RNA-seq that had HER2 RNA expression levels available as transcripts per million (TPM) were used to validate RNAscope. Briefly, an RNA library was prepared using the Ribo-zero kit (Illumina, Inc. San Diego, CA, USA) and then paired-end sequenced using a NovaSeq 6000 (Illumina, Inc.). Each dataset was filtered to remove reads that were aligned using Spliced Transcripts Alignment to a Reference (STAR) to the human genome reference Genome Reference Consortium Human Build 38 (GRCh38) transcript annotations from the UCSC knownGenes table. Gene and transcript levels were quantified using RNA-Seq by Expectation-Maximization (RSEM) (version 1.2.31).

### Statistical analysis

Data processing, analyses, and plotting were performed using GraphPad Prism 6 and 9 (GraphPad Software Inc., San Diego, CA, USA) and IBM SPSS Statistics for Windows version 19 (IBM, Armonk, NY, USA). The kappa coefficient was used to assess interobserver agreement for HER2 RNAscope score evaluation. Pearson’s Chi-square or Fisher’s exact tests, as appropriate, were used to compare categorical variables. Statistical significance of the differential expression of HER2 was determined using an unpaired two-tailed t-test. A backward stepwise logistic regression model was used to evaluate the effects of covariates on pCR. If a variable remained at *p* < 0.15, it was included in the multivariate model [27]. Statistical significance was set at *p* value < 0.05.

## Results

### HER2 RNAscope scoring as a validated semiquantitative analysis of isHRE

**Fig. 1:**
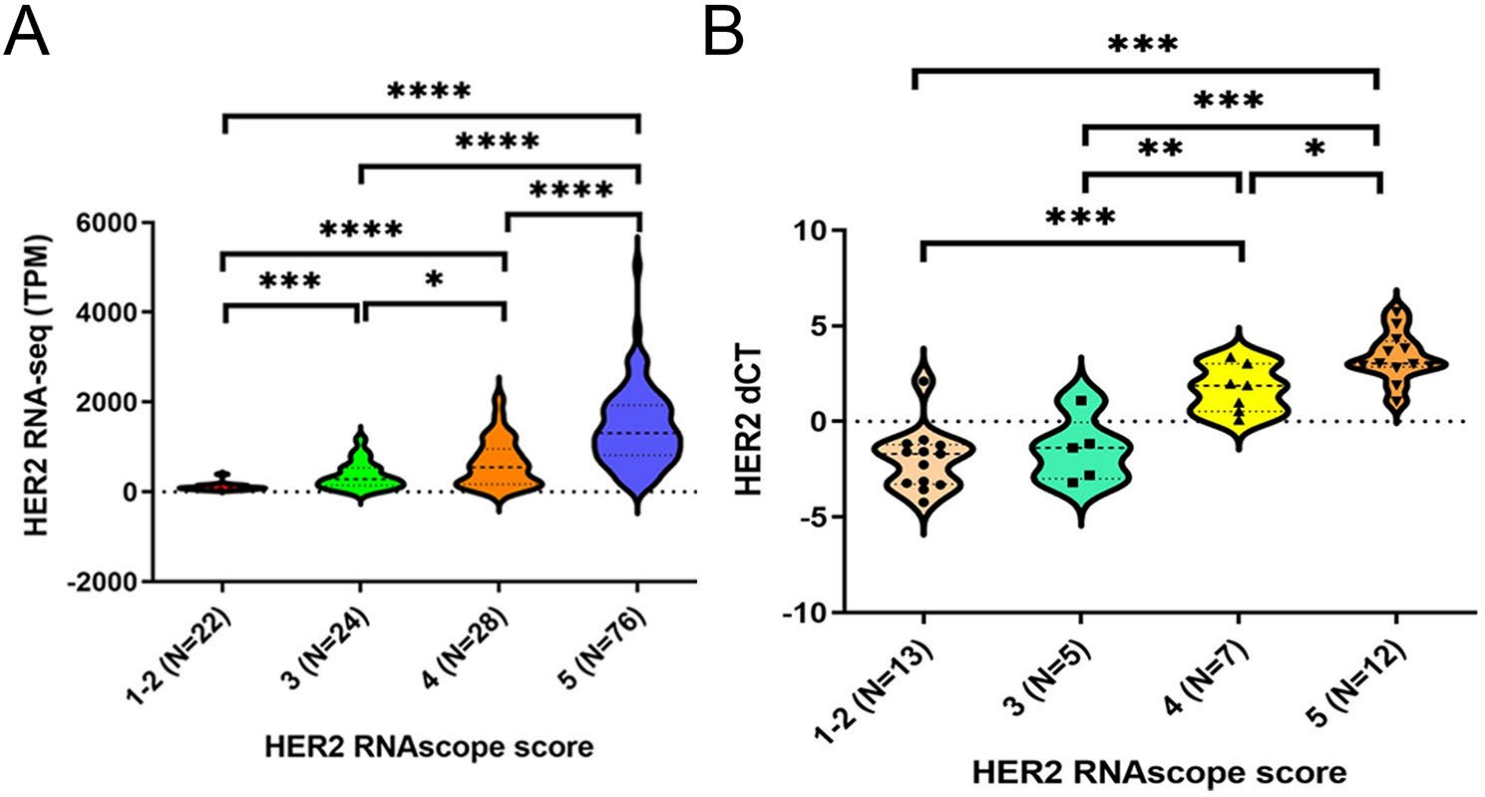
Validation of HER2 RNAscope scoring as a semiquantitative method to evaluate the isHRE. Violin plots denoting the transcripts per million (TPM) value of HER2 gene expression by RNA-seq (**A**) and the dCT value by qRT-PCR (**B**) among BCs with HER2 RNAscope scores 1–2, 3, 4, and 5. dCT was calculated as (CtGAPDH – CtHER2). *p < 0.05, **p < 0.01, ***p < 0.001, and ****p < 0.0001

To determine whether HER2 RNAscope scoring could be used for the semiquantitative analysis of isHRE, we initially evaluated the correlation between HER2 RNAscope scores and HER2 RNA expression determined by RNA-seq in 150 BC cases. We observed significantly higher HER2 RNA expression in patients with scores of 5 versus 4, 4 versus 3, and 3 versus 1 and 2 (1–2), respectively (Fig. 1A). Similarly, HER2 RNA expression determined by qRT-PCR was shown as dCT (CtGAPDH – CtHER2) in additional 37 BCs ranging from HER2 IHC 0 to 3 + and was significantly higher in cases with a score of 5 versus 4 and 4 versus 3 (Fig. 1B). However, the difference between the scores of 3 and 1–2, did not reach statistical significance. These findings demonstrated an overall positive correlation between HER2 RNAscope scores and RNA expression levels, validating RNAscope scoring as a semiquantitative method for evaluating isHRE.

### Patients characteristics

Among the 278 HER2-positive BCs used for HER2 RNAscope, 43 cases had scores ≤ 2 and received further RNAscope for PPIB. Four patients had a PPIB score of 1 and were considered to have degraded RNA. Four cases showed clear clonal (zonal) heterogeneous RNAscope signals. These eight cases were excluded from subsequent analysis. A total of 270 patients were included for further analysis. The kappa coefficient of the HER2 RNAscope scores for the 270 cases between the two reviewers was 0.808 (*p* < 0.001). Details of the clinicopathological and treatment features are summarized (Supplementary Table S2). HR was positive and negative in 50.4% and 49.6% of the patients, respectively. HER2 IHC 3 + and 2+/FISH + were observed in 79.3% and 20.7% of the patients, respectively. Mono (H) and dual (H and P) anti-HER2 blockade was performed in 46.3% and 53.7% of the patients, respectively. The overall pCR rate was 40.7%.

### Positive correlation of isHRE with pCR rates

**Fig. 2:**
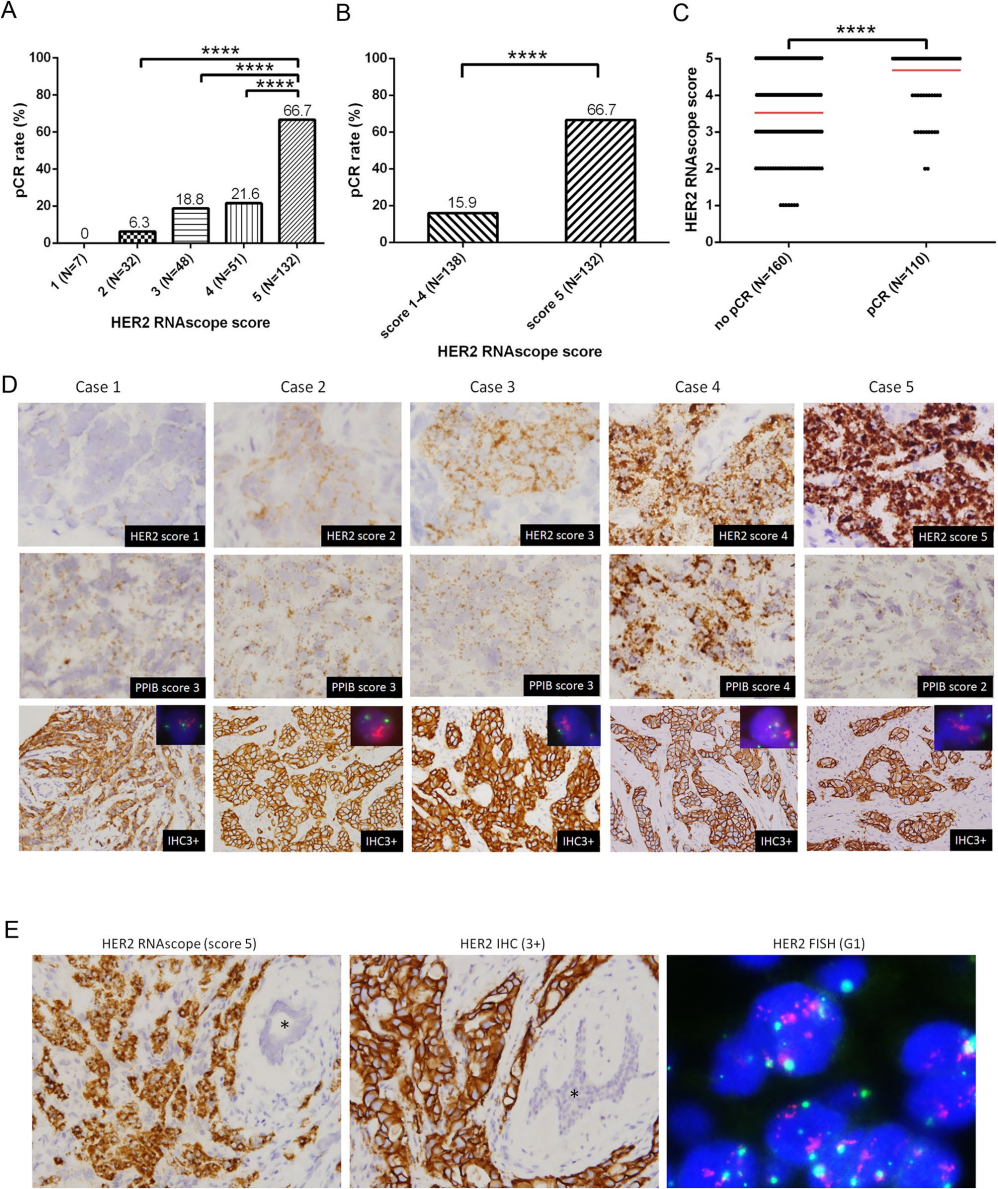
Correlation between HER2 RNAscope scores and pCR rates in the 270 patients with HER2-positive BCs receiving NCTT, and representative images of HER2 and PPIB RNAscope scores with corresponding HER2 IHC and FISH. Barplots of the pCR rates in patients stratified by HER2 RNAscope scores 1–5 (**A**) and 1–4 versus 5 (**B**). (**C**) Dotplot denoting HER2 RNAscope scores in patients with or without pCR. Mean is shown as red line. (**D**) Representative BCs with HER2 RNAscope score 1, 2, 3, 4 or 5 (upper panel), with corresponding control PPIB RNAscope scores (middle panel), HER2 IHC (lower panel) and HER2 FISH (inset in lower panel). (**E**) A representative case of HER2 RNAscope (left panel), IHC (middle panel) and FISH (right panel) is shown. Star, non-neoplastic mammary duct. ****p < 0.0001

To investigate the effect of isHRE on anti-HER2 therapeutic efficacy, we analyzed the relationship between HER2 RNAscope scores and pCR rates in 270 patients with HER2-positive BC who received NCTT. The pCR rate was 66.7% in patients with BCs with a score of 5, which was significantly higher than the rates of 0%, 6.3%, 18.8%, and 21.6% in patients with BCs with scores of 1, 2, 3, and 4, respectively (Fig. 2A). The difference in pCR rates was significantly higher in cases with a score of 5 versus scores of 1–4 (66.7% versus 15.9%, *p* < 0.0001) (Fig. 2B). Conversely, patients who achieved pCR had significantly higher scores than those without pCR (Fig. 2C). Representative images demonstrating HER2 RNAscope scoring and the corresponding IHC and FISH results are shown (Fig. 2D and E). This finding demonstrated a positive correlation between HER2 RNAscope scores and pCR rates, with significantly higher pCR rates in patients with BCs with scores of 5 versus score 1–4.

### Positive impact of isHRE on pCR rates in HER2-positive IHC/FISH categories

**Fig. 3:**
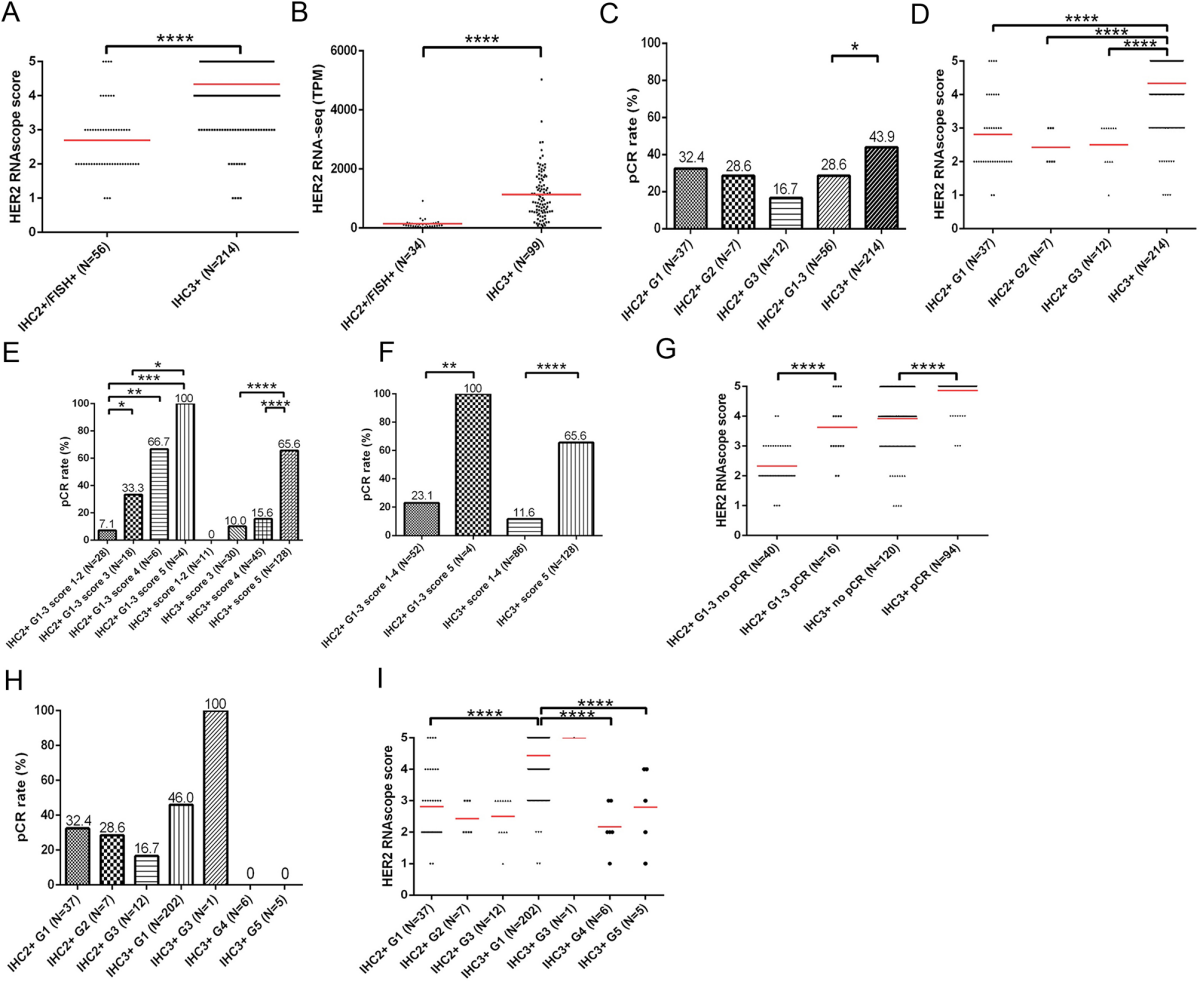
Correlation of HER2 RNAscope scores and pCR rates among HER2 IHC/FISH categories in the 270 patients with HER2-positive BCs receiving NCTT. Dotplots denoting the disctribution of HER2 RNAscope scores (**A**) and HER2 RNA-seq TPM (**B**) among the two HER2-positive BC groups. Barplot and dotplot denoting the pCR rates (**C**) and HER2 RNAscope scores (**D**), respectively, among each HER2-positive IHC/FISH category. Barplots denoting the pCR rates among HER2-positive IHC/FISH categories stratified by HER2 RNAscope scores (1–2, 3, 4, and 5) (**E**) or (1–4 and 5) (**F**). **G** Dotplot showing HER2 RNAscope scores in patients with or without pCR among the HER2-positive IHC/FISH categories. Barplot and dotplot denoting the pCR rates (**H**) and HER2 RNAscope scores (**I**), respectively, among cases with IHC 2 + or IHC 3 + stratified by FISH result. Mean is shown in red line. *p < 0.05, **p < 0.01, ***p < 0.001, and ****p < 0.0001

To assess the HER2 RNA expression in HER2 IHC 3 + and 2+/FISH + BCs, the two HER2-positive IHC/FISH categories, we analyzed their HER2 RNAscope scores and TPM values. We observed significantly higher RNAscope scores and TPM values in HER2 IHC 3 + cells than in IHC 2+/FISH + BCs (Fig. 3A and B). Notably, a wide range of RNAscope scores and TPM values was observed, particularly in HER2 IHC 3 + BCs, suggesting heterogeneous HER2 RNA expression even within the same HER2-positive IHC category. Next, we investigated the effects of isHRE on pCR rates in various HER2-positive IHC/FISH categories. The pCR rate was significantly higher in patients with IHC 3 + than in those with IHC 2+/FISH + BCs (Fig. 3C), and the RNAscope score was significantly higher in patients with IHC 3 + than in those with IHC 2+/FISH G1-3 BCs (Fig. 3D and Supplementary Table S3). When patients were stratified by RNAscope scores, we observed a positive correlation between pCR rates and RNAscope scores in both IHC categories (Fig. 3E), with the difference in pCR rates particularly evident when cases were stratified by scores of 5 and scores of 1–4 (Fig. 3F). Reciprocally, patients who achieved pCR had significantly higher HER2 RNAscope scores in both IHC categories (Fig. 3G). Because all 214 HER2 3 + BCs samples underwent additional FISH tests, we further investigated the pCR rates and RNAscope scores among the various FISH groups in both IHC categories. The pCR rates and RNAscope scores were numerically higher in patients with BC with IHC 3+/FISH G1 and G3 than in those with IHC 2+/FISH G1 and G3 (Fig. 3H and I). Patients with IHC 3+/FISH G4 and G5 had significantly lower RNAscope scores than those with IHC 3+/FISH G1, and none of these patients achieved pCR. These results demonstrated the positive effect of isHRE on pCR rates within the HER2-positive IHC/FISH categories.

### isHRE and pCR rates showing similar non-linear positive correlation with both HER2 copy numbers and HER2/CEP 17 copy number ratios

**Fig. 4:**
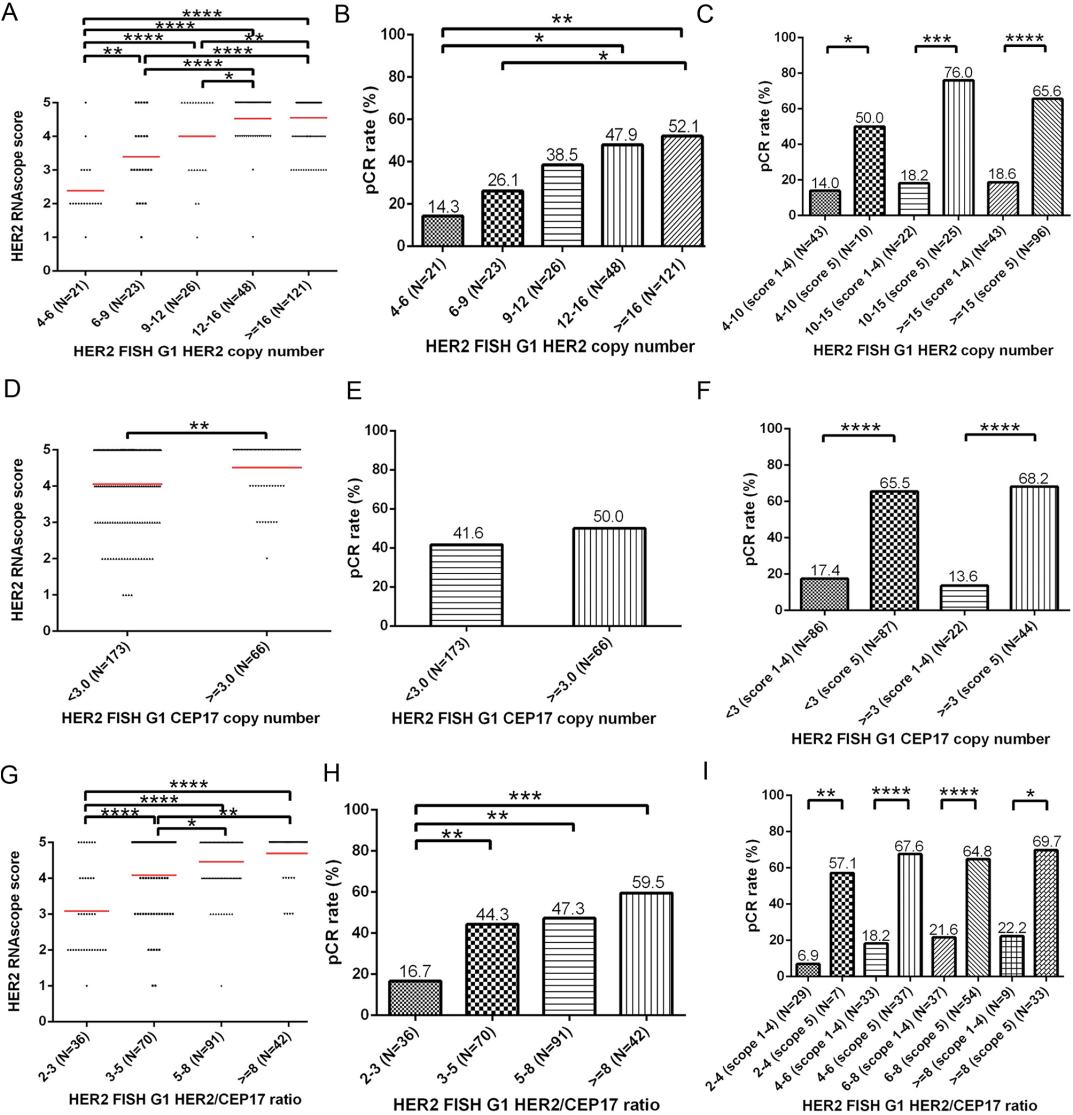
Correlation of HER2 RNAscope scores and pCR rates with HER2 copy numbers, CEP17 copy numbers and HER2/CEP17 copy number ratios in the 239 patients with HER2 FISH G1 BCs. Dotplot and barplot denoting the HER2 RNAscope scores (**A**) and pCR rates (**B**), respectively, among BC cases stratified by HER2 copy numbers in the range of 4–6 ( ≧ 4, < 6), 6–9, 9–12, 12–16, and ≧ 16. **C** Barplot showing the pCR rates of BC cases stratified by HER2 RNAscope scores (1–4 versus 5) within each HER2 copy number range. Dotplot and barplot denoting the HER2 RNAscope scores (**D**) and pCR rates (**E**), respectively, among BC cases stratified by CEP17 copy number of 3. **F** Barplot showing the pCR rates of BC cases stratified by HER2 RNAscope scores (1–4 versus 5) in BC cases with CEP17 copy number < 3 or ≧ 3. Dotplot and barplot denoting the HER2 RNAscope scores (**G**) and pCR rates (**H**), respectively, among BC cases stratified by HER2/CEP17 copy number ratios in the range of 2–3 ( ≧ 2, < 3), 3–5, 5–8, and ≧ 8. **I** Barplot showing the pCR rates of BC cases stratified by HER2 RNAscope scores (1–4 versus 5) within each HER2/CEP17 copy number ratio range. *p < 0.05, **p < 0.01, ***p < 0.001, and ****p < 0.0001

Next, we investigated the correlation of HER2 FISH parameters, namely HER2 and CEP17 copy numbers and HER2/CEP17 copy number ratios, with isHRE and pCR rates in all 239 HER2 FISH G1 cases. We observed a non-linear positive correlation between HER2 RNAscope scores and HER2 copy numbers: with scores significantly lower in cases with copy number 4–6 ( ≧ 4, < 6) versus 6–9, but not significantly different between those with copy number 12–16 and ≧ 16 (Fig. 4A). The correlation of HER2 copy number groups with pCR rates showed a pattern similar to that of the HER2 RNAscope scores (Fig. 4B). Consistently, we observed significantly higher pCR rates in BC cases with RNAscope scores of 5 versus 1–4, among all HER2 copy number categories (Fig. 4C). CEP17 amplification, defined as CEP17 copy number ≧ 3 [23], correlated with higher RNAscope scores (Fig. 4D), but corresponding pCR rates were not significantly different between those with or without CEP17 amplification (Fig. 4E). However, an RNAscope score of 5 significantly correlated with higher pCR rates, both in cases with and without CEP17 amplification (Fig. 4F). The correlation of both RNAscope scores and pCR rates with HER2/CEP17 copy number ratios showed patterns similar to those of HER2 copy numbers (Fig. 4G-H). Similarly, significantly higher pCR rates were observed in cases with a score of 5 versus scores of 1–4 in all four ratio groups (Fig. 4I). Together, these findings demonstrate the positive impact of isHRE on the non-linear positive correlation between pCR rates and HER2 copy numbers as well as HER2/CEP17 copy number ratios.

### Positive impact of isHRE on pCR rates within HR categories

**Fig. 5:**
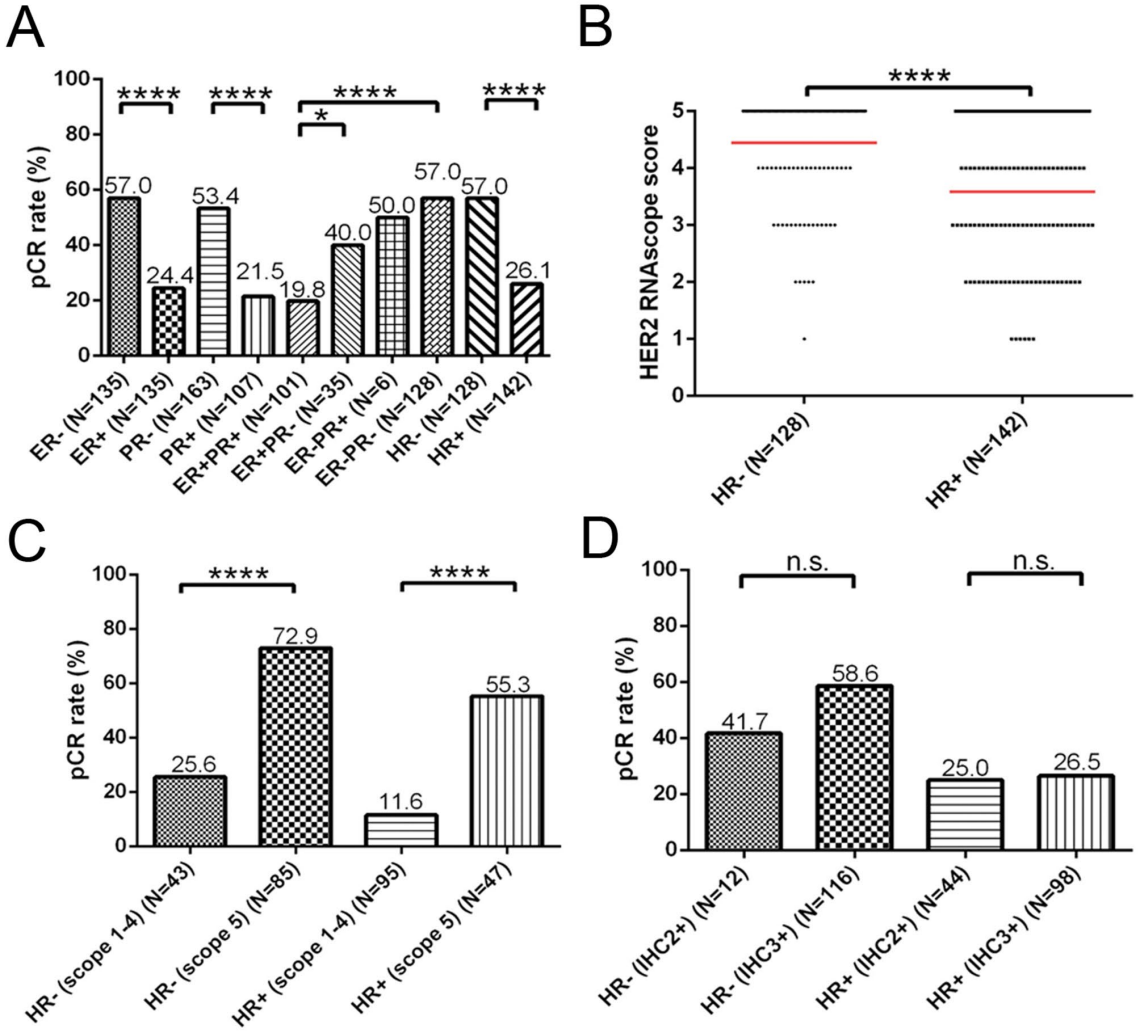
Correlation of pCR rates and HER2 RNAscope scores within HR categories in the 270 patients with HER2-positive BCs receiving NCTT. (**A**) Barplot denoting the pCR rates among various HR status, with HR + defined as ER + and/or PR+, and HR- as ER- and PR-. (**B**) Dotplot denoting the HER2 RNAscope scores among HR- and HR + BCs. Barplot denoting the pCR rates among HR- and HR + BCs patients stratified by HER2 RNAscope scores (score 1–4 versus 5) (**C**) or by HER2 IHC (IHC 2+/FISH + versus 3+) (**D**). n.s., not significant. *p < 0.05, **p < 0.01, ***p < 0.001, and ****p < 0.0001

Next, we investigated the effect of isHRE on pCR rates within the HR categories. Patients with BCs negative for ER, PR, or HR had significantly higher pCR rates than those with BCs positive for ER, PR, or HR, with the highest and lowest pCR rates in patients with ER/PR double-negative and double-positive BCs, respectively, supporting the negative impact of HR on pCR rates (Fig. 5A). We further observed significantly higher HER2 RNAscope scores in HR- versus HR + BCs (Fig. 5B), and significantly higher pCR rate in cases with RNAscope score 5 versus score 1–4 BCs in both HR+ (55.3% vs. 11.6%, *p* < 0.0001) and HR- (72.9% vs. 25.6%, *p* < 0.0001) cases (Fig. 5C). Compared with HER2 RNAscope scoring, HER2 status determined by HER2 IHC/FISH did not stratify HR + or HR- BC patients, with a significant difference in the pCR rates (Fig. 5D). Together, these findings demonstrated the positive effect of isHRE on pCR rates in both HR- and HR + BC patients.

### Factors associated with pCR on multivariate analysis and their combinational impact on the pCR rates

Univariate and multivariate analyses were performed to identify the clinicopathological factors associated with pCR. The results are summarized in Table 1. In multivariate analysis, an HER2 RNAscope score of 5 remained a significant and strong pCR predictor [odds ratio (OR) = 10.865, *p* < 0.001]. HR (OR = 1.907, *p* = 0.045), TIL ≧ 20% (OR = 2.057, *p* = 0.031), and anti-HER2 dual blockade (OR = 2.178, *p* = 0.018) were also significantly associated with higher pCR rates. We further investigated the combined effect of HER2 RNAscope, HR, and anti-HER2 blockade on pCR rates (Table 2). Overall, patients with HR-/score 5 BCs receiving dual blockade (dual/HR-/score 5) had the highest pCR rate (80.8%), compared to the much lower pCR rates of 10.3%, 12.5%, and 16.7% in patients with mono/HR+/score 1–4, dual/HR+/score 1–4 and mono/HR-/score 1–4 BCs, respectively. Cases with HR- BCs had significantly higher pCR rates than those with HR + BCs when receiving anti-HER2 dual blockade (64.9% vs. 22.1%, *p* < 0.001). Compared to patients receiving anti-HER2 mono blockade, patients receiving anti-HER2 dual blockade had significantly higher pCR rates only for HR- BCs (64.9% vs. 45.1%, *p* = 0.030), or score 5 BCs (72.9% vs. 55.3%, *p* = 0.040), or HR-/score 5 BCs (80.8% vs. 60.6%, *p* = 0.041). In contrast, cases with a score of 5 BCs had significantly higher pCR rates than those with score of 1–4 BCs irresspective of HR or anti-HER2 blockade status (all *p* < 0.003). In contrast to RNAscope scores of 5 versus 1–4, patients with BC HER2 status determined by IHC 3 + versus IHC 2+/FISH + did not have significantly different pCR rates, regardless of the combined status of HR and anti-HER2 blockade. These findings supported a HER2 RNAscope score of 5 as a significant and strong predictor of pCR.

**Table 1:** Univariate and multivariate logistic regression model for pCR according to clinicopathological and treatment factors

Factors				pCR			
		Univariate analysis			Multivariate analysis		
		OR	95% CI	*p* value	OR	95% CI	*p* value
Age < 50y vs. ≧50y		1.307	0.798–2.141	0.287			
cT1-2 vs. cT3		0.759	0.368–1.566	0.456			
cN0 vs. cN1-3		0.726	0.410–1.285	0.272			
Grade I-II vs. III		1.418	0.871–2.310	0.160			
HR positive vs. negative		3.767	2.256–6.290	**< 0.001**	1.907	1.014–3.584	**0.045**
HER2 RNAscope score 1–4 vs. 5		10.545	5.893–18.872	**< 0.001**	10.865	5.547–21.281	**< 0.001**
TIL < 20% vs. ≧20%		2.082	1.226–3.534	**0.007**	2.057	1.068–3.961	**0.031**
NCTT subgroup							
	EC(F) to TH(P) vs. TH(P)	2.307	1.049–5.072	**0.038**	2.680	0.941–7.634	0.065
	EC(F) to TH(P) vs. TH(P) to EC(F)	1.004	0.518–1.948	0.990	1.363	0.599–3.099	0.460
	EC(F) to TH(P) vs. TCarboH(P)	1.405	0.700-2.819	0.339	1.096	0.435–2.764	0.846
Anti-HER2 blockade (mono vs. dual)		1.444	0.885–2.359	0.142	2.178	1.141–4.159	**0.018**

Bold font indicates statistical significance at the *p* < 0.05 level

**Table 2:** Impact of combination of multivariate analysis-derived pCR predictors on pCR rates of the 270 patients with HER2-positive BCs receiving NCTT

			pCR								
		Negativity	Positivity	Total	*p* value						
IHC											
	2+ / FISH+	40 (71.4)	16 (28.6)	56 (100)	**0.047**						
	3+	120 (56.1)	94 (43.9)	214 (100)							
HER2 RNAscope score											
	1–4	116 (84.1)	22 (15.9)	138 (100)	**< 0.001**						
	5	44 (33.3)	88 (66.7)	132 (100)							
HR											
	positive	105 (73.9)	37 (26.1)	142 (100)	**< 0.001**						
	negative	55 (43.0)	73 (57.0)	128 (100)							
anti-HER2 blockade											
	mono	80 (64.0)	45 (36.0)	125 (100)	0.172						
	dual	80 (55.2)	65 (44.8)	145 (100)							
HR plus HER2 RNAscope score											
	HR+ / score 1–4	84 (88.4)	11 (11.6)	95 (100)	**< 0.001** ^**1**^	**0.037** ^**3**^					
	HR+ / score 5	21 (44.7)	26 (55.3)	47 (100)			**0.040** ^**4**^				
	HR- / score 1–4	32 (74.4)	11 (25.6)	43 (100)	**< 0.001** ^**2**^						
	HR- / score 5	23 (27.1)	62 (72.9)	85 (100)							
HR plus anti-HER2 blockade											
	HR+ / mono	52 (70.3)	22 (29.7)	74 (100)	0.342	0.090					
	HR+ /dual	53 (77.9)	15 (22.1)	68 (100)			**< 0.001**				
	HR- / mono	28 (54.9)	23 (45.1)	51 (100)	**0.030**						
	HR- / dual	27 (35.1)	50 (64.9)	77 (100)							
HER2 RNAscope score plus anti-HER2 blockade											
	1–4 / mono	50 (87.7)	7 (12.3)	57 (100)	0.356	**< 0.001**					
	1–4 / dual	66 (81.5)	15 (18.5)	81 (100)			**< 0.001**				
	5 / mono	30 (44.1)	38 (55.9)	68 (100)	**0.009**						
	5 / dual	14 (21.9)	50 (78.1)	64 (100)							
anti-HER2 blockade plus HR plus HER2 RNAscope											
	mono / HR+ / 1–4	35 (89.7)	4 (10.3)	39 (100)	**< 0.001**	0.667		0.737^5^			
	mono / HR+ / 5	17 (48.6)	18 (51.4)	35 (100)			0.474		0.360^6^		
	mono / HR- / 1–4	15 (83.3)	3 (16.7)	18 (100)	**0.003**					0.256^7^	
	mono / HR- / 5	13 (39.4)	20 (60.6)	33 (100)							**0.041** ^**8**^
	dual / HR+ / 1–4	49 (87.5)	7 (12.5)	56 (100)	**< 0.001**	0.061					
	dual / HR+ / 5	4 (33.3)	8 (66.7)	12 (100)			0.438				
	dual / HR- / 1–4	17 (68.0)	8 (32.0)	25 (100)	**< 0.001**						
	dual / HR- / 5	10 (19.2)	42 (80.8)	52 (100)							
anti-HER2 blockade plus HR plus IHC											
	mono / HR+ / IHC 2+	15 (68.2)	7 (31.8)	22 (100)	0.788	1.000		0.488			
	mono / HR+ / IHC 3+	37 (71.2)	15 (28.8)	52 (100)			0.092		0.650		
	mono / HR- / IHC 2+	4 (66.7)	2 (33.3)	6 (100)	0.678					1.000	
	mono / HR- / IHC 3+	24 (53.3)	21 (46.7)	45 (100)							0.053
	dual / HR+ / IHC 2+	18 (81.8)	4 (18.2)	22 (100)	0.758	0.144					
	dual / HR+ / IHC 3+	35 (76.1)	11 (23.9)	46 (100)			**< 0.001**				
	dual / HR- / IHC 2+	3 (50.0)	3 (50.0)	6 (100)	0.659						
	dual / HR- / IHC 3+	24 (33.8)	47 (66.2)	71 (100)							

*p* value of ^1^ HR+ / score 1–4 vs HR+ / score 5; ^2^ HR- / score 1–4 vs HR- / score 5; ^3^ HR+ / score 1–4 vs HR- / score 1–4; ^4^ HR+ / score 5 vs HR- / score 5; ^5^ mono / HR+ / 1–4 vs dual / HR+ /1–4; ^6^ mono / HR+ / 5 vs dual / HR+ / 5; ^7^ mono / HR- / 1–4 vs dual / HR- / 1–4; ^8^ mono / HR- / 5 vs dual /HR- / 5

Bold font indicates statistical significance at the *p* < 0.05 level

### Positive impact of isHRE on the pCR rates within the NCTT treatment subgroups

**Fig. 6:**
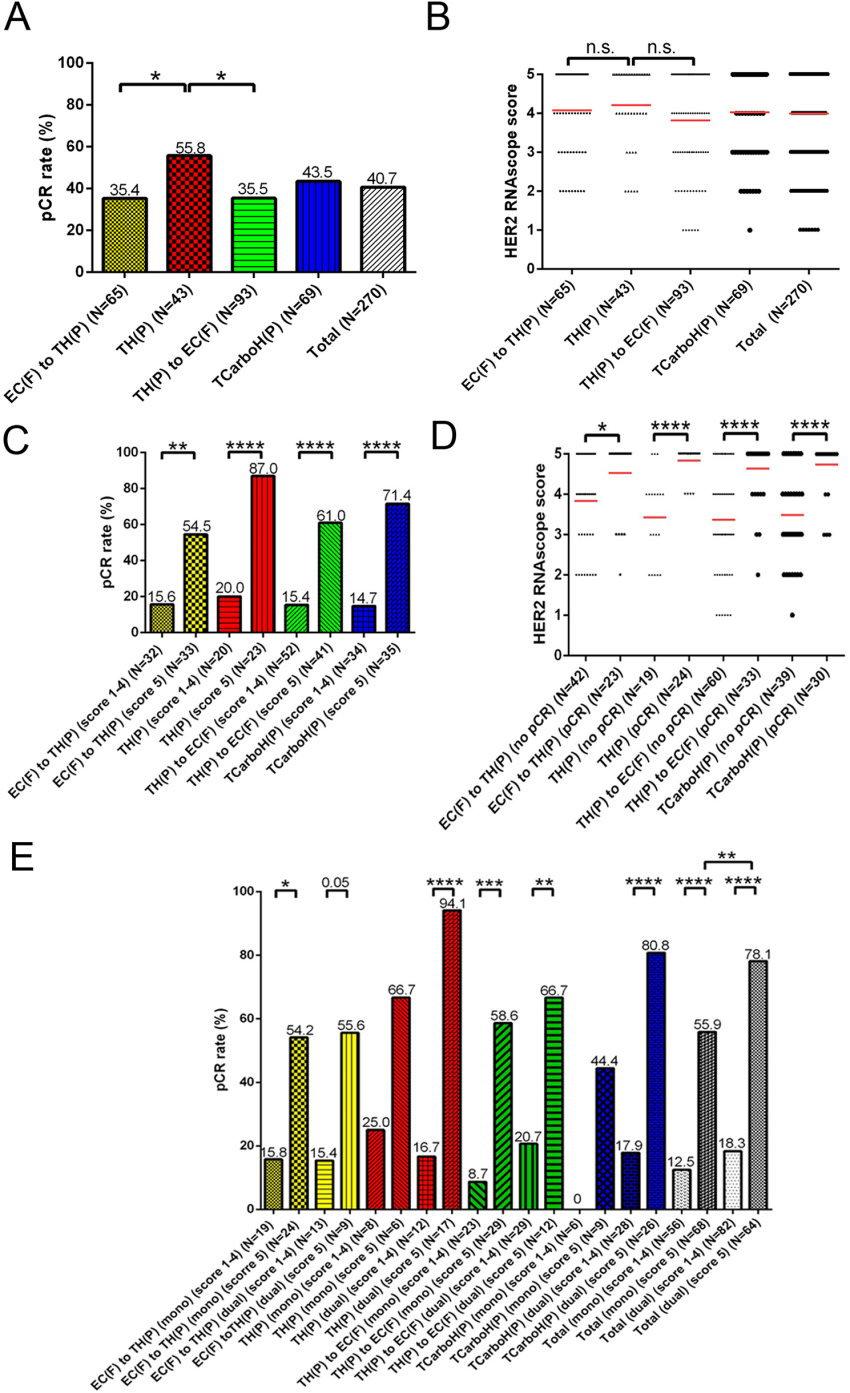
The pCR rates and the impact of HER2 RNAscope score and anti-HER2 mono/dual blockade on the pCR rates on the four NCTT treatment patient subgroups. Barplot and dotplot denoting the pCR rates (**A**) and HER2 RNAscope scores (**B**), respectively, among the four NCTT subgroups. Barplot and dotplot showing the pCR rates (**C**) and HER2 RNAscope scores (**D**) of BC cases among patients of the four NCTT subgroups. (**C**) is stratified by HER2 RNAscope score while (**D**) is stratified by pCR. (**E**) Barplot denoting the pCR rates of patients stratified by HER2 RNAscope (score 1–4 versus 5) and anti-HER2 mono/dual blockade. *p < 0.05, **p < 0.01, ***p < 0.001, and ****p < 0.0001

Finally, we investigated the effect of isHRE on pCR rates in the NCTT treatment subgroups. We observed significantly higher pCR rates in patients receiving TH(P) versus EC(F) to TH(P) (55.8% vs. 35.4%, *p* = 0.048) or TH(P) to EC(F) (55.8% vs. 35.5%, *p* = 0.039) (Fig. 6A), however, the HER2 RNAscope scores were not significantly different between these subgroups (Fig. 6B), suggesting that the difference in the pCR rates was not attributed to isHRE. Nevertheless, within each NCTT subgroup, significantly higher pCR rates were observed in patients with a score of 5 versus 1–4 BCs (Fig. 6C), and patients achieving pCR had higher scores than those without (Fig. 6D), which is consistent with the impact of isHRE on pCR rates. Because patients in the TH(P) subgroup had higher rates of anti-HER2 dual blockade (Supplementary Table S4), we further stratified the patients based on the status of anti-HER2 blockade and HER2 RNAscope scores. In all four NCTT subgroups, we observed consistently higher pCR rates in patients with a score of 5 versus score of 1–4 BCs irrespective of the anti-HER2 blockade status (Fig. 6E). In contrast, a significantly higher pCR rate was observed in patients receiving dual versus mono anti-HER2 blockade only when they had BCs with a score of 5 (78.1% vs. 55.9%, *p* = 0.009). These findings confirmed the positive impact of isHRE on pCR rates in all four NCTT subgroups and demonstrated that the impact of dual versus mono blockade may depend on the isHRE status.

## Discussion

In this study, we demonstrated that RNAscope scoring can be applied as a semiquantitative method to evaluate the HER2 RNA expression status in tissues. We showed the isHRE among the HER2-positive IHC/FISH categories and the correlation of isHRE with various HER2 FISH parameters. We demonstrated that HER2 RNAscope scoring, particularly using the two-tier score of 1–4 versus 5, could be used to stratify HER2 expression status by standard HER2 IHC/FISH and showed that such stratification was highly correlated with pCR rates. Moreover, when HER2 RNAscope scoring was classified from five tiers (scores 1–5) to two tiers (scores 1–4 versus 5), the kappa interobserver agreement score increased from 0.808 to 0.911 (both *p* < 0.001). Our results demonstrated that HER2 RNAscope scoring can be applied as a useful, convenient, reproducible, and tissue-related diagnostic adjunct to standard HER2 biomarkers to further stratify HER2 expression status. This stratification is highly correlated with the efficacy of anti-HER2 treatment in patients with HER2-positive BCs receiving NATT.

Herein, we demonstrated a positive correlation between isHRE and pCR rates in patients with HER2-positive BCs receiving NCTT, providing in situ evidence confirming the role of HER2 RNA levels as a key determinant of pCR [5]. The particularly significant difference in pCR rates between patients BCs with score of 1–4 versus 5 supports the finding that an RNAscope score of 5 is a strong indicator of pCR. In line with previous reports [7, 8, 20, 28], we demonstrated higher pCR rates in cases with IHC 3 + versus IHC 2+/FISH + BCs, with significantly higher HER2 RNAscope scores in the former, suggesting that isHRE may underlie the differences in pCR rates. Furthermore, we observed a wide range of HER2 RNA expression, as determined by RNAscope and RNA-seq, in HER2-positive BCs, particularly in the IHC 3 + HER2 category. This finding, together with the positive correlation between HER2 RNAscope scores and pCR rates, underscores the role of RNAscope scoring in the further stratification of HER2 expression status in HER2-positive BCs, because such stratification is related to therapeutic efficacy. Moreover, such stratification may also help identify patients with conventional IHC/ISH-defined HER2-positive BCs with lower isHRE who may potentially benefit from alternative HER2-targeted treatments targeting low-HER2 BCs [29].

The effect of HER2 FISH categories on anti-HER2 therapeutic efficacy remains controversial [7, 8, 11–13]. In this study, patients with HER2 IHC 3+/FISH G1 and G3 BCs were associated with higher pCR rates and higher HER2 RNAscope scores than those with IHC 2+ /FISH G1 and G3 BCs, suggesting that, given the same FISH groups, HER2 RNA and protein levels may determine pCR rates. Moreover, the RNAscope scores and pCR rates were relatively lower in patients with IHC 2+/FISH G3 than in those with G1 BCs. These findings, while supporting the inclusion of FISH G3 with IHC 3 + as HER2 positivity, generally revealed lower isHRE and pCR rates in patients with IHC 2+/FISH G3 BCs. In contrast, although IHC 2+/FISH G2 was no longer considered HER2 FISH-positive, the isHRE was comparable between IHC 2+/FISH G2 and G3 BCs, and patients with IHC 2+/FISH G2 BCs achieved a pCR rate of 28.6%, which is comparable to the rate of 27% reported by Rakha et al. [8] and numerically higher than that in IHC 2+/FISH G3 cases. Given the comparable isHREs between IHC 2+/FISH G2 and G3 BCs, more cases should be evaluated for eligibility for anti-HER2 therapy in these rare HER2 2+/FISH categories.

The impact of HER2 FISH parameters on anti-HER2 therapeutic efficacy remains controversial [7–16]. Herein, we demonstrated that isHRE and pCR rates showed similar nonlinear positive correlations with both HER2 copy numbers and HER2/CEP 17 copy number ratios. Our findings reaffirmed the impact of HER2 copy number and HER2/CEP17 ratio on anti-HER2 therapeutic efficacy [7, 9, 10, 15, 16], and demonstrated the positive impact of isHRE on the non-linear positive correlation between pCR rates and HER2 copy numbers as well as HER2/CEP17 copy number ratios. The effect of CEP17 amplification on HER2 alterations and pCR rates remains controversial [14, 30–34]. Although we observed that CEP17 amplification correlated with slightly higher HER2 RNAscope scores, pCR rates were not significantly different between patients with and without CEP17 amplification, arguing against its impact on anti-HER2 efficacy in a neoadjuvant setting.

HR status influences the pCR rates in neoadjuvant setting with anti-HER2 therapeutic efficacy [20, 35–37]. Herein, we demonstrated HR negativity as an indicator of higher pCR rates in multivariate analysis, consistent with the positive impact of HR negativity on anti-HER2 therapeutic efficacy [7, 38]. Moreover, we showed that, compared to HR + BCs, HR- BCs had significantly higher HER2 RNAscope scores and demonstrated that a high HER2 RNAscope score (score 5) correlated with significantly higher pCR rates both in patients with HR- and HR + BCs. Notably, the pCR rates were not significantly different between immunohistochemical staining of HER2 2 + and 3 + patients with either HR + or HR– BCs. Our findings suggested a positive impact of isHRE on anti-HER2 therapeutic efficacy in patients with both HR + and HR- BC and demonstrated the role of RNA ISH in better stratifying HER2 status in both HR + and HR- BCs, which is related to anti-HER2 neoadjuvant therapeutic efficacy. TIL levels ≧ 20% was also demonstrated as a pCR predictor in multivariate analysis in the present study, compatible with previous reports showing a positive impact of TIL in pCR rates in patients with HER2-positive BCs [26].

The effect of the combination of HR and anti-HER2 mono/dual blockade with isHRE on anti-HER2 therapeutic efficacy remains unexplored. In this study, we found that patients with HR-/score 5 BCs receiving dual blockade had the highest pCR rate of 80.8%, in sharp contrast to the lowest rate of 10.3% in patients with HR+/score 1–4 BCs receiving monoblockade (Table 2). This finding suggested that the combined impact of these factors may be beneficial in predicting the responses to NCTT. In addition, patients with a BC score of 5 had significantly higher pCR rates than those with score of 1–4 irrespective of their HR, HER2 blockade status, and NCTT treatment subgroups. In contrast, patients with HR- BCs had significantly higher pCR rates than those with HR + BCs only on those receiving dual anti-HER2 blockade; patients receiving dual anti-HER2 blockade had significantly higher pCR rates only on HR- BCs or BCs with a score of 5. Together, these findings support high isHRE as a significant and strong predictor of pCR. Notably, compared to isHRE (score 5 versus 1–4), HER2 status determined by IHC (IHC 3 + versus IHC 2+/FISH+) did not significantly differ in pCR rates between patients stratified by HR and HER2 blockade status. Our findings reaffirm the impact of HER2 RNA expression on anti-HER2 treatment efficacy [5] and highlight the role of isHRE in better stratification of HER2 expression status, which is relevant to anti-HER2 therapeutic efficacy.

In conclusion, we demonstrated that HER2 RNAscope scoring is a valid, convenient, and reproducible method to semiquantitatively determine HER2 RNA expression in tissues. We showed a positive impact of isHRE, particularly HER2 RNAscope score 5, on the pCR rates in patients with BCs among the HER2-positive IHC/FISH, HR, and NCTT treatment categories, and demonstrated a positive impact of isHRE on the non-linear positive correlation between pCR rates and HER2 copy numbers as well as HER2/CEP17 ratios. The combined impact of multivariate pCR predictors supported the isHRE of an RNAscope score of 5 as a significant and strong predictor of pCR. Our results reaffirmed the critical role of HER2 RNA expression in anti-HER2 therapeutic efficacy and also highlighted the complementary role of isHRE to standard HER2 biomarkers in stratifying HER2 status in tissues, particularly using a cutoff of score 1–4 versus a score of 5, and such stratification is clinically relevant.

### Electronic supplementary material

Below is the link to the electronic supplementary material.

Supplementary Material 1

## Data Availability

The data are available from the corresponding author upon reasonable request.

## Abbreviations

ASCO: American Society of Clinical Oncology
BC: Breast cancer
Carbo: Carboplatin
CAP: College of American Pathologists
CEP17: Centromeric 17
Ct: Cycle threshold
C: Cyclophosphamide
DAB: 3,3’-diaminobenzidine
E: Epirubicin
ER: Estrogen receptor
F: Fluorouracil
FFPE: Formalin-fixed, paraffin-embedded
FISH: Fluorescence in situ hybridization
GAPDH: Glyceraldehyde-3-phosphate dehydrogenase
GRCh38: Genome Reference Consortium Human Build 38
G: Group
H: Herceptin
HER2: Human epidermal growth factor 2
HR: Hormone receptor
IHC: Immunohistochemistry
ISH: In situ hybridization
isHRE: In situ HER2 RNA expression
NCTT: Neoadjuvant chemotherapy and anti-HER2 targeted treatment
OR: Odds ratio
pCR: Pathological complete response
PPIB: Peptidylprolyl isomerase B
P: Pertuxumab
PR: Progesterone receptor
qRT-PCR: Quantitative reverse transcriptase polymerase chain reaction
RNA-seq: Rna sequencing
RSEM: RNA-Seq by Expectation-Maximization
STAR: Spliced Transcripts Alignment to a Reference
T: Taxotere or taxol
TPM: Transcripts per million
TIL: Tumor infiltrating lymphocyte
