# Supplementary material for: In situ HER2 RNA expression as a predictor of pathologic complete response of HER2-positive breast cancer patients receiving neoadjuvant chemotherapy and anti-HER2 targeted treatment
Source: Breast Cancer Res. 2024 Jun 12;26:100. doi: 10.1186/s13058-024-01852-3 (PMC11170871; doi:10.1186/s13058-024-01852-3)
Supplement: Supplementary file 1 — Supplementary Material 1 [file 13058_2024_1852_MOESM1_ESM.pdf]

Supplementary Information

Supplementary Table S1. Primer sequences used in qRT-PCR

Supplementary Table S2. Demography of the 270 patients with HER2-positive BCs receiving NCTT

Supplementary Table S3. The HER2 RNAscope scores of 270 breast cancer cases were categorized using HER2 IHC and FISH.

Supplementary Table S4. Clinicopathological factors in the four NCTT treatment subgroups

Supplementary Table S1. Primer sequences used in qRT-PCR

Gene	Forward sequence	Reverse sequence
HER2	ACAGT GGCAT CTGTG AGCTG	CCCAC GTCCG TAGAA AGGTA
GAPDH	GAGTC AACGG ATTTG GTCGT	GACAA GCTTC CCGTT CTCAG

Supplementary Table S2. Demography of the 270 patients with HER2-positive BCs receiving NCTT

			n (%)
Age			
	<50y		116 (43.0)
	≧50y		154 (57.0)
Histology subtype			
	NST		264 (97.8)
	non-NST		6 (2.2)
SBR grade			
	I		1 (0.4)
	II		143 (53.0)
	III		126 (46.7)
Pre-NCTT			
	cT		
		cT1	25 (9.2)
		cT2	208 (77.0)
		cT3	37 (13.7)
	cN		

	cN0		62 (23.0)
	cN1-3		208 (77.0)
Post-NCTT			
ypT			
	ypT0		77 (28.5)
	ypTis		41 (15.2)
	ypT1		
		ypT1mic	14 (5.2)
		ypT1a	26 (9.6)
		ypT1b	24 (8.9)
		ypT1c	24 (8.9)
	ypT2		53 (19.6)
	ypT3		11 (4.1)
ypN			
	ypN0		146 (54.1)
	ypN1-3		85 (31.5)
	ypNx		39 (14.4)
Hormone receptor (HR)			
ER			

		positivity		136 (50.4)
		negativity		134 (49.6)
	PR			
		positivity		107 (39.6)
		negativity		163 (60.4)
	ER/PR			
		HR positivity		
			ER+/PR+	101 (37.4)
			ER+/PR-	35 (13.0)
			ER-/PR+	6 (2.2)
		HR negativity		
			ER-/PR-	128 (47.4)
HER2				
	IHC2+/FISH+			
		G1		37 (13.7)
		G2		7 (2.6)
		G3		12 (4.4)
	IHC3+			214 (79.3)
Post-NCTT operation				

Partial mastectomy	133 (49.3)
Total mastectomy	137 (50.7)
Post-NCTT lymph node dissection	
SLND	61 (22.6)
ALND	170 (63.0)
Not performed	39 (14.4)
NCTT regimen	
EC(F) to TH(P)	65 (24.1)
TH(P)	43 (15.9)
TH(P) to EC(F)	93 (34.4)
TCarboH(P)	69 (25.6)
Anti-HER2 blockade	
mono	125 (46.3)
dual	145 (53.7)
Pathologic complete response	
Yes	110 (40.7)
No	160 (59.3)

Supplementary Table S3. The HER2 RNAscope scores of 270 breast cancer cases were categorized using HER2 IHC and FISH.

			RNAscope score					
		1	2	3	4	5	Total	*p* value
HER2 IHC / FISH								
IHC 2+	FISH G1	2 (5.4)	17 (45.9)	8 (21.6)	6 (16.2)	4 (10.8)	37 (100)	0.238
	FISH G2	0 (0)	4 (57.1)	3 (42.9)	0 (0)	0 (0)	7 (100)	
	FISH G3	1 (8.3)	4 (33.3)	7 (58.3)	0 (0)	0 (0)	12 (100)	
IHC 3+	FISH G1	2 (1.0)	3 (1.5)	27 (13.4)	43 (21.3)	127 (62.9)	202 (100)	**< 0.001**
	FISH G3	0 (0)	0 (0)	0 (0)	0 (0)	1 (100)	1 (100)	
	FISH G4	1 (16.7)	3 (50.0)	2 (33.3)	0 (0)	0 (0)	6 (100)	
	FISH G5	1 (20.0)	1 (20.0)	1 (20.0)	2 (40.0)	0 (0)	5 (100)	

The Pearson chi-square test is employed to assess the association between individual FISH groups in cases of HER2 IHC 2+ or 3+ and HER2 RNA scores. The *p*-value is calculated using the chi-square test.

Bold font indicates statistical significance at the $p < 0.05$ level.

Supplementary Table 4. Clinicopathological factors in the four NCTT treatment subgroups

Factors		EC(F) to TH(P)	TH(P)	TH(P) to EC(F)	TCarboH(P)	*p* value
Age						0.222
	< 50y	31 (47.7)	14 (32.6)	45 (48.4)	26 (37.4)	
	≧ 50y	34 (52.3)	29 (67.4)	48 (51.6)	42 (62.3)	
Histology subtype						0.183
	NST	64 (98.5)	41 (95.3)	93 (100)	66 (95.7)	
	non-NST	1 (1.5)	2 (4.7)	0 (0)	3 (4.3)	
SBR grade						0.777
	I/II	35 (53.8)	20 (46.5)	50 (53.8)	39 (56.5)	
	III	30 (46.2)	23 (53.5)	43 (46.2)	30 (43.5)	

Pre-NAC							
	cT						0.884
		cT1/T2	58 (89.2)	37 (86.0)	79 (84.9)	59 (85.5)	
		cT3/T4	7 (10.8)	6 (14.0)	14 (15.1)	10 (14.5)	
	cN						0.221
		cN0	12 (18.5)	6 (14.0)	26 (28.0)	18 (26.1)	
		cN1-3	53 (81.5)	37 (86.0)	67 (72.0)	51 (73.9)	
HR							0.165
	Negativity		31 (47.7)	23 (53.5)	36 (38.7)	38 (55.1)	
	Positivity		34 (52.3)	20 (46.5)	57 (61.3)	31 (44.9)	
HER2 IHC / FISH							0.661

	IHC 2+ / FISH+	16 (24.6)	9 (20.9)	20 (21.5)	11 (15.9)	
	IHC 3+	49 (75.4)	34 (79.1)	73 (78.5)	58 (84.1)	
HER2 RNAscope score						0.704
	score 1-4	32 (49.2)	20 (46.5)	52 (55.9)	34 (49.3)	
	score 5	33 (50.8)	23 (53.5)	41 (44.1)	35 (50.7)	
Anti-HER2 blockade						**< 0.001**
	mono	43 (66.2)	14 (32.6)	53 (57.0)	15 (21.7)	
	dual	22 (33.8)	29 (67.4)	40 (43.0)	54 (78.3)	

The Pearson chi-square test is employed to assess the association between the clinicopathological factors and the four NCTT treatment subgroups. The *p*-value is calculated using the chi-square test.

Bold font indicates statistical significance at the $p < 0.05$ level.
